# Inequity in Continuous Glucose Monitor (CGM) Prescribing Behaviors in Primary Care

**DOI:** 10.1007/s11606-025-09923-7

**Published:** 2026-01-13

**Authors:** Jovan Milosavljevic, Clyde Schechter, Melissa Fazzari, Priyanka Mathias, Justin Mathew, Shivani Agarwal

**Affiliations:** 1 Fleischer Institute for Diabetes and Metabolism, Division of Endocrinology, Albert Einstein College of Medicine and Montefiore Medical Center, Bronx, NY USA; 2 Department of Family and Social Medicine, Albert Einstein College of Medicine and Montefiore Medical Center, Bronx, NY USA; 3 Department of Epidemiology and Population Health, Albert Einstein College of Medicine, Bronx, NY USA; 4 Community Health Improvement & Strategy, Network Performance Group, Montefiore Medical Center, Bronx, NY USA; 5 Network Performance Group, Montefiore Medical Center, Yonkers, NY USA

**Keywords:** primary care, continuous glucose monitoring, diabetes technology, inequity

## Abstract

**Background:**

Continuous glucose monitoring (CGM) has become a standard of care for diabetes management, yet its adoption in primary care remains limited where the majority of people with diabetes are served. Examining contributors to CGM prescriptions in primary care may identify new targets for intervention that could improve diabetes population health.

**Objective:**

To determine factors associated with CGM prescription by primary care providers (PCPs) in a safety-net primary care practice network serving over 11,000 adults with insulin-treated type 2 diabetes.

**Design:**

Retrospective cohort study.

**Patients:**

Adults with type 2 diabetes treated with insulin with at least one primary care visit between July 31, 2020, and July 31, 2023, in a safety-net primary care clinic network in the Bronx, NY.

**Main Measures:**

Primary outcome was first-time CGM prescription by PCPs. Multivariable analysis was performed using cause-specific Cox regression, with death and prescriptions by endocrinology treated as censoring events.

**Results:**

Among 11,037 insulin-treated patients (mean age 61 ± 14 years; 56% female; 44% Hispanic, 39% non-Hispanic Black), 17% received first-time CGM prescriptions from primary care, with a median monthly prescription rate of 3.1% (2.8–3.3%). Older patients, Spanish-speaking, and publicly insured had a lower likelihood of receiving CGM prescription. Conversely, patients with higher HbA1c levels, more intensive insulin regimens, and providers with more years in practice were more likely to receive CGM regardless of race-ethnicity.

**Conclusions:**

CGM prescribing rates in primary care remain low, with inequity that favored younger, English-speaking, commercially insured patients, and more experienced providers. We have highlighted gaps in workflow efficiency, language and age-appropriate training support, and trainee education as key next steps to address in order to increase CGM prescriptions in primary care practice. Addressing inequitable CGM prescribing patterns is a critical first step to elevating the standard of diabetes care in primary care practice and breaking the cascade of inequity in outcomes for underserved diabetes populations.

**Supplementary Information:**

The online version contains supplementary material available at 10.1007/s11606-025-09923-7.

## BACKGROUND

There is a critical need to increase the use of continuous glucose monitoring (CGM) for monitoring of insulin-treated patients in primary care, given its potential to transform diabetes management and improve patient outcomes on a population level. CGM use has been shown to reduce HbA1c levels^1^, hypoglycemia^2^ and improve quality of life,^3^ representing a powerful tool to improve population-level outcomes in diabetes. As such, the American Diabetes Association recommends CGM as the modality for glucose monitoring for all individuals with any type of diabetes on insulin therapy.^4^ Recently, accessibility for CGM has increased with expanded insurance coverage by Medicaid and Medicare.^5^ Coupled with additional financial reimbursement benefits for in-clinic placement and interpretation of glycemic results,^6^ CGM offers an important tool to improve diabetes care while enabling a financial model for sustainability.

The majority of outpatient diabetes visits occur in primary care.^7,8^ Additionally, primary care patients are more likely to be of minority race/ethnicity and to have type 2 diabetes, compared to patients seen by specialists.^7^ Thus, adopting CGM in primary care will extend the reach of these technologies to a larger part of the diabetes population. With the national shortage of endocrinologists^9^ and rising diabetes incidence,^10^ primary care will remain and will increasingly become the only point of care for most people with diabetes. Nevertheless, CGM adoption in primary care remains low.^11^ Among 30,585 adults with type 2 diabetes in primary care at a large academic medical center, only 5% were prescribed CGM, compared to 25% of those who also saw an endocrinologist.^12^ Similarly, in a cohort of 2,578 adults with type 2 diabetes enrolled in Colorado Medicaid, 12% received a CGM prescription in primary care, compared to 69% under endocrinology care.^13^ Increased attention and effort must be paid to increase appropriate CGM use in primary care practice. Moreover, inequities in CGM prescribing within endocrinology settings are well-documented, disproportionately affecting racial-ethnic minority groups,^14,15^ older adults,^16^ non-English speakers,^13^ and publicly insured patients,^17^ and it is critical to investigate whether these disparities extend into primary care. While prior studies evaluated CGM use across all prescribers or focused on specialty settings, the determinants of PCP-initiated CGM remain poorly defined. 

We aimed to examine first-time PCP-initiated prescriptions among insulin-treated, coverage-eligible adults, delineating patient- and provider-level drivers of uptake in a large urban academic health system with ready access to endocrinology, a focus that supports scalable CGM expansion through primary care even where specialists are available. We hypothesized that CGM prescriptions in primary care practices are affected by a combination of patient and provider characteristics, and will identify new targets for interventions.

## METHODS

### Study Design and Subjects

This is a retrospective cohort study of patients with type 2 diabetes seen in a large primary care clinic network at Montefiore Medical Center (MMC) between July 31, 2020, and July 31, 2023. MMC is a large safety-net hospital system in the Bronx, NY, the most diverse (55% Hispanic, 30% non-Hispanic Black) and underserved county with the highest diabetes prevalence in New York state.^18,19^ CGM is covered under Medicaid and Medicaid-managed insurance plans in New York state for patients with type 2 diabetes on any insulin,^20^ which mitigates insurance barriers as a reason for not having CGM prescribed.

Patients with type 2 diabetes were identified using ICD-10 codes and included if they were ≥ 18 years old and had at least one clinical encounter with a primary care provider during the study period. Additionally, we included only patients with an existing insulin prescription to focus on those meeting current clinical practice recommendations for CGM eligibility. Patients were excluded from analysis if they were not eligible for CGM prescription, i.e., did not have an in-person or telemedicine visit during the study period, did not have insurance, or had a CGM prescription prior to the study start date.

The baseline date was defined as July 31, 2020, or the date of the first insulin prescription if it was after July 31, 2020. Patients were followed until the earliest of the following events: the date of the first CGM prescription, the date of death, or the censoring date of July 31, 2023.

### Measures

The primary outcome was the first-time CGM prescription by a primary care provider. We identified primary care providers by using the National Provider Identifier (NPI) database and verifying their specialty. CGM prescriptions included Dexcom G4/G5/G6/G7 or FreeStyle Libre 1/2/3. Covariates included baseline demographics (age, sex, race-ethnicity, self-reported preferred spoken language), insurance type (commercial, Medicaid, Medicare, dual Medicaid-Medicare), presence of macrovascular or microvascular diabetes complications (cardiovascular, cerebrovascular, nephropathy, peripheral vascular disease, neuropathy, or retinopathy), hypoglycemia, treatment regimen (basal insulin only, multiple daily insulin), longitudinal HbA1c levels, hospitalizations, endocrinology visits, primary care office visit frequency, clinic site, and primary care provider years in practice (years since NPI enumeration date). Complications were identified using ICD‑10 codes. Nephropathy was defined with any of (1) ICD-10 code for diabetic nephropathy, (2) ≥ 2 urine microalbumin-creatinine ratios > 30 mg/g, or (3) ≥ 2 eGFR measurements < 60 mL/min/1.73 m^2^ taken at least 90 days apart. To establish a clear temporal relationship between predictors and CGM prescription, covariates were only included if they occurred before the initial CGM prescription. All clinical data were obtained through the electronic health record (EHR).

### Statistical Analysis

Baseline characteristics were represented as mean ± standard deviation (SD), or median and interquartile range (IQR) for continuous variables, and frequencies and percentages for categorical variables.

Monthly CGM prescription rates were calculated by dividing the number of insulin-treated patients with type 2 diabetes who received a first CGM prescription from their primary care provider during a given month by the total number of patients who had a primary care office visit that month and no history of prior CGM use.

Cause-specific Cox regression was used to model the incidence of CGM prescription by primary care providers. Censoring occurred for patients at the time of their first CGM prescription by a non-primary care provider (e.g., endocrinology), at the time of death during the study period, or at the end of the study period (July 31, 2023) if CGM had not been prescribed by that time. Time-fixed covariates included age, sex, insurance status, preferred language, PCP years in practice, and clinic site. Time-varying covariates included diagnosis of microvascular and macrovascular complications, insulin treatment regimen, HbA1c levels, hospitalization events, and office visit count. To address missing HbA1c levels, we used multiple imputation by chained equations. The details about the multiple imputation and sensitivity analyses are provided in the Supplement (eMethods, Supplemental Table 3). Adjusted hazard ratios (HR) with 95% confidence intervals (95% CI) are reported. Two-sided *p* values < 0.05 were considered statistically significant. Analyses were performed using R version 4.3.2 (R Foundation for Statistical Computing) and Stata version 18.0 (StataCorp LLC).

## RESULTS

We identified a total of 53,636 patients with type 2 diabetes who had at least one clinical encounter during the study period. After excluding patients without an insulin prescription (*n* = 28,146), patients without at least one office or telemedicine visit at selected primary care sites (*n* = 12,845), patients without insurance (*n* = 134), and patients with an existing CGM prescription before the study start (*n* = 1474), the final cohort included 11,037 patients eligible for new CGM. These patients received care from a network of 1,259 primary care providers.

Baseline characteristics of the study population are listed in Table 1. For this insulin-treated cohort, mean ± SD age was 61 ± 14 years; 56% female; 44% Hispanic, 39% Non-Hispanic Black (NHB); 40% Medicare, 29% Medicaid, 10% dual Medicare-Medicaid.

**Table 1:** Baseline Patient and Provider Characteristics: Overall and by CGM Prescription Status

**Variable**^a^	**Insulin-treated patients** (*n* = 11,037)^b^	**No CGM prescription** (*n* = 8,213)^b^	**CGM prescribed by PCP** (*n* = 1,874)^b^	**CGM prescribed by non-PCP** (*n* = 950)^b^
***Patient characteristics***				
** Age (years)**	61.3 ± 14.2	62.1 ± 14.1	59.9 ± 13.4	56.9 ± 15.2
** Sex**				
Female	6,212 (56.3)	4,585 (55.8)	1,063 (56.7)	564 (59.4)
Male	4,825 (43.7)	3,628 (44.2)	811 (43.3)	386 (40.6)
**Race-ethnicity**				
Non-Hispanic White	448 (4.1)	342 (4.2)	69 (3.7)	37 (3.9)
Hispanic	4,819 (43.7)	3,599 (43.8)	786 (41.9)	434 (45.7)
Non-Hispanic Black	4,290 (38.9)	3,123 (38.0)	797 (42.5)	370 (38.9)
Asian/Pacific	384 (3.5)	300 (3.7)	53 (2.8)	31 (3.3)
Other	1,096 (9.9)	849 (10.3)	169 (9.0)	78 (8.2)
**Preferred language**				
English	8,337 (75.5)	6,038 (73.5)	1,523 (81.3)	776 (81.7)
Spanish	2,329 (21.1)	1,877 (22.9)	302 (16.1)	150 (15.8)
Other	371 (3.4)	298 (3.6)	49 (2.6)	24 (2.5)
**Insurance**				
Commercial	2,312 (20.9)	1,580 (19.2)	498 (26.6)	234 (24.6)
Medicare	4,374 (39.6)	3,330 (40.5)	701 (37.4)	343 (36.1)
Medicaid	3,191 (28.9)	2,414 (29.4)	490 (26.1)	287 (30.2)
Dual Medicare-Medicaid	1,160 (10.5)	889 (10.8)	185 (9.9)	86 (9.1)
**Diabetes complications**				
Any microvascular^c^	6,988 (63.3)	5,157 (62.8)	1,194 (63.7)	637 (67.1)
Any macrovascular^d^	4,765 (43.2)	3,591 (43.7)	772 (41.2)	402 (42.3)
Hypoglycemia^e^	1,330 (12.1)	938 (11.4)	211 (11.3)	181 (19.1)
**Hemoglobin A1c (%)** (*n* = 8647)^f^	9.3 ± 2.5	9.1 ± 2.5	9.8 ± 2.4	9.5 ± 2.4
**Insulin regimen**				
Long-acting insulin	9,974 (90.4)	7,363 (89.7)	1,734 (92.5)	877 (92.3)
Rapid-acting insulin	5,245 (47.5)	3,615 (44.0)	971 (51.8)	659 (69.4)
Short-acting insulin	47 (0.4)	31 (0.4)	8 (0.4)	8 (0.8)
Insulin NPH	295 (2.7)	228 (2.8)	52 (2.8)	15 (1.6)
Premixed insulin	472 (4.3)	322 (3.9)	100 (5.3)	50 (5.3)
**Number of primary care office visits in last 12 months**	2 (0–4)	2 (0–4)	2 (0–3)	2 (0–3)
**Any hospitalization in last 12 months**	3,693 (33.5)	2,730 (33.2)	578 (30.8)	385 (40.5)
**Endocrinology visit in last 12 months**	976 (8.8)	545 (6.6)	83 (4.4)	348 (36.6)
***Primary care provider characteristics***				
**PCP years in practice** (*n* = 10,985)				
3 years or less	910 (8.3)	832 (10.2)	74 (3.9)	4 (0.4)
3 to 10 years	3,275 (29.8)	2,237 (27.4)	547 (29.2)	491 (51.7)
10 years or more	6,800 (61.9)	5,092 (62.4)	1,253 (66.9)	455 (47.9)
**PCP clinic** (*n* = 10,985)				
Family medicine	2,004 (18.3)	1,454 (17.8)	415 (22.2)	135 (14.4)
Internal medicine	8,969 (81.7)	6,707 (82.2)	1,457 (77.8)	805 (85.6)

The table summarizes characteristics at baseline date, defined as the date of first insulin prescription (or July 31, 2020, for those already on insulin). No patient had a CGM prescription at entry. All group comparisons were statistically significant at *p* < 0.05, except for age, macrovascular complications, use of short‑acting insulin, and use of insulin NPH, ^a^Abbreviations: *CGM*, continuous glucose monitor; *PCP*, primary care provider; *NPH*, neutral protamine Hagedorn, ^b^Numerical data expressed as mean ± SD or median (IQR). Categorical data expressed as *n* (%), ^c^Diagnosed neuropathy, retinopathy, or nephropathy prior to baseline date, ^d^Diagnosed cardiovascular, peripheral vascular, or cerebrovascular disease prior to baseline date, ^e^Hypoglycemia was identified using ICD-10 diagnosis codes that capture both diabetes-related hypoglycemia and unspecified hypoglycemia recorded prior to baseline date, ^f^Last hemoglobin A1c within 1 year prior to baseline date

Over 3 years of follow-up, a total of 1,874 (16.9%) insulin-treated patients with diabetes received first-time CGM prescriptions by primary care providers. The median monthly new CGM prescription rate was 3.1% (IQR 2.8–3.3) (Supplemental Fig. 1). Patients who had CGM prescribed over the study period were younger, more often English-speaking, and commercially insured, and had higher baseline HbA1c levels compared to those who were not prescribed CGM (Table 1). Characteristics at the time of CGM prescription are shown in Table 2, and changes from baseline to the end of follow-up are summarized in Supplemental Table 2.

**Table 2:** Patient Characteristics at End of Follow-up: Overall and by CGM Prescription Status

**Variable**^a^	**Insulin-treated patients** (*n* = 11,037)^b^	**No CGM** (*n* = 8,213)^b^	**CGM by PCP** (*n* = 1,874)^b^	**CGM by non-PCP** (*n* = 950)^b^
**Diabetes complications**				
Any microvascular^c^	8,011 (72.6)	5,978 (72.8)	1,324 (70.7)	709 (74.6)
Any macrovascular^d^	5,859 (53.1)	4,475 (54.5)	914 (48.8)	470 (49.5)
Hypoglycemia event^e^	2,073 (18.8)	1,531 (18.6)	290 (15.5)	252 (26.5)
**Hemoglobin A1c** (%) (*n* = 10,836)^f^	8.5 ± 2.3	8.1 ± 2.2	9.7 ± 2.3	9.5 ± 2.3
**Insulin regimen**				
Long-acting insulin	10,408 (94.3)	7,688 (93.6)	1,800 (96.1)	920 (96.8)
Rapid-acting insulin	6.041 (54.7)	4.187 (51.0)	1.109 (59.2)	745 (78.4)
Short-acting insulin	104 (0.9)	70 (0.9)	19 (1.0)	15 (1.6)
Insulin NPH	387 (3.5)	298 (3.6)	65 (3.5)	24 (2.5)
Premixed insulin	583 (5.3)	409 (5.0)	112 (6.0)	62 (6.5)
**Endocrinology visit during follow-up**	2,862 (25.9)	1,512 (18.4)	486 (25.9)	864 (90.9)

The table shows patient characteristics that changed over study follow-up time. Characteristics shown in this table were recorded on the date of first CGM prescription for those who initiated CGM, or on the study end date for those without CGM prescription. All group comparisons were statistically significant at *p* < 0.05, except for insulin NPH. Baseline-to-follow-up changes for each characteristic are provided in Table S2, ^a^Abbreviations: *CGM*, continuous glucose monitor; *PCP*, primary care provider; *NPH*, neutral protamine Hagedorn, ^b^Numerical data expressed as mean ± SD or median (IQR). Categorical data expressed as *n* (%), ^c^Diagnosed neuropathy, retinopathy, or nephropathy during follow-up, ^d^Diagnosed cardiovascular, peripheral vascular, or cerebrovascular disease during follow-up, ^e^Any hypoglycemia event during follow-up, ^f^Last known hemoglobin A1c level during study follow-up

**Figure 1:**
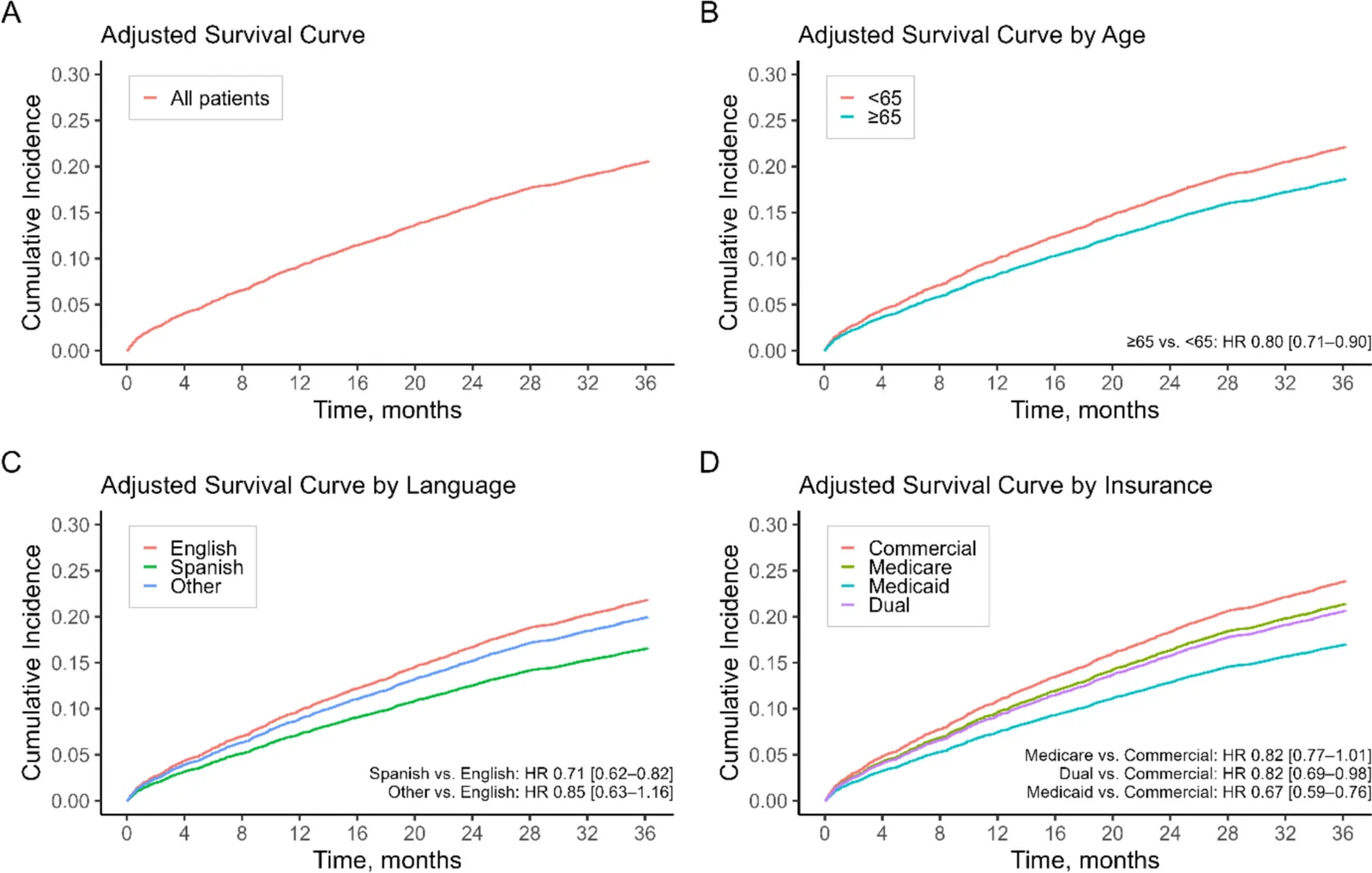
Time to continuous glucose monitor prescription by primary care. Cause-specific adjusted survival curves for time to first continuous glucose monitor prescription issued by a primary care provider overall (**A**), by age (**B**), preferred language (**C**), and insurance status (**D**). Survival curves were estimated from the Cox proportional hazards model shown in Table 3. Prescriptions by non-primary care and deaths were treated as censoring events. Curves are adjusted for all covariates in the model, including demographics, clinical factors, and provider characteristics. Each panel displays the corresponding adjusted hazard ratio (HR) with its 95% confidence interval (CI).

Figure 1 shows the adjusted survival curves showing time to the first CGM prescription by primary care. Results of the multivariable analysis are shown in Table 3. The following describes factors where CGM was less likely to be prescribed. CGM was 21% less likely to be prescribed for patients older vs. younger than 65 years (HR 0.79 (0.70–0.90)) and 29% less likely for Spanish- vs. English-speaking (HR 0.71 (0.62–0.82)). Compared to commercially insured, patients were 33% less likely to be prescribed CGM if on Medicaid (HR 0.67 (0.59–0.76)), 28% less likely if dual-eligible for Medicaid-Medicare (HR 0.82 (0.69–0.98)), and 22% less likely if on Medicare (HR 0.88 (0.77–1.01)). Lastly, patients were 17% less likely to be prescribed CGM if receiving care by internal medicine vs. family medicine providers (HR 0.83 (0.74–0.93)).

**Table 3:** Factors Influencing CGM Prescription by Primary Care Providers

Characteristic*	Adjusted HR	95% CI
Age		
< 65 years	—	—
≥ 65 years	0.80	0.71 to 0.90
Sex		
Female	—	—
Male	0.97	0.88 to 1.06
Race-ethnicity		
Non-Hispanic White	—	—
Hispanic	1.02	0.79 to 1.31
Non-Hispanic Black	1.02	0.79 to 1.31
Asian/Pacific	0.84	0.58 to 1.21
Other	0.97	0.73 to 1.29
Preferred language		
English	—	—
Spanish	0.71	0.62 to 0.82
Other	0.85	0.63 to 1.16
Insurance		
Commercial	—	—
Medicare	0.88	0.77 to 1.01
Dual Medicare-Medicaid	0.82	0.69 to 0.98
Medicaid	0.67	0.59 to 0.76
Any microvascular complication (yes vs. no)	1.01	0.91 to 1.13
Any macrovascular complication (yes vs. no)	1.00	0.91 to 1.11
Hypoglycemia event (yes vs. no)	1.02	0.89 to 1.16
Insulin regimen		
Basal insulin only	—	—
MDI insulin	1.40	1.27 to 1.54
Hemoglobin A1c (per each %-pt increase)	1.23	1.20 to 1.25
Hospitalization during follow-up	1.12	0.90 to 1.40
Primary care office visit count (per each visit)	1.07	1.06 to 1.08
Endocrinology co-management	1.11	1.01 to 1.24
PCP years in practice (per each year)	1.03	1.02 to 1.04
PCP clinic site		
Internal medicine	—	—
Family medicine	0.83	0.74 to 0.93

^*^Abbreviations: *CGM*, continuous glucose monitor; *MDI*, multiple daily injection insulin; *PCP*, primary care provider; *IM*, internal medicine; *FM*, family medicine, HR and 95% CI were calculated using cause-specific Cox regression (*n* = 1,872 events). Patients were censored at the time of first-time CGM prescription by non-primary care provider (*n* = 950, 8%) and death (*n* = 349, 3%) or at the end of follow-up. Time-varying covariates included microvascular and macrovascular complications, hypoglycemia events, insulin regimen changes, hemoglobin A1c levels, hospitalization events, and primary care visit counts. Microvascular complications were defined as either neuropathy, retinopathy, or nephropathy. Macrovascular complications were defined as cardiovascular, cerebrovascular, or peripheral vascular disease. Multiple imputation by chained equations was used to address longitudinal missing hemoglobin A1c levels (overall missingness 3.8%)

The following describes factors where CGM was more likely to be prescribed. CGM was 41% more likely to be prescribed for those on a multiple daily injection (MDI) insulin regimen vs. basal only insulin (HR 1.41 (1.28–1.53)), 23% more likely with each additional HbA1c percentage point increase (HR 1.23 (1.20–1.25)), 7% more likely with each additional primary care office visit (HR 1.07 (1.06–1.08)), and 3% more likely with each additional prescriber year in practice (HR 1.03 (1.02–1.04)). Lastly, patients with an endocrinology visit during study follow-up time were 11% more likely to receive CGM prescription from their PCP, compared to patients who were managed by primary care alone (HR 1.11 (1.01–1.24)).

Sex, race-ethnicity, presence of diabetes complications, and hypoglycemia events were not significantly associated with CGM prescription rates.

## DISCUSSION

Our study characterized CGM prescribing behaviors of 1,259 primary care providers in a large New York safety-net hospital system caring for over 11,000 adults with type 2 diabetes. We found very low prescription rates among eligible, insulin-treated patients, despite diabetes technology being widely covered by public insurance in our state. Additionally, we found that patients with higher HbA1c levels and on intensive insulin regimens were more likely to receive CGM prescriptions regardless of race-ethnicity, whereas patients who were on Medicaid or were dual-eligible, Spanish-speaking, or older were less likely. We also found that patients seen by providers with more years of practice were more likely to receive the prescription. Since CGM prescription is the first step toward patient use, addressing differential prescribing rates at this proximal step may help reduce disparities in subsequent uptake and CGM-associated clinical outcomes.

We were surprised to see inequity in prescription by public versus private insurance given that New York state has generous diabetes technology coverage. Notably, we found that insurance type remained a significant predictor of CGM prescription even after adjusting for race-ethnicity, while race-ethnicity itself was not significant. This pattern points towards policy and system-level barriers perceived with public insurance, rather than implicit racial or ethnic bias. A recent American Diabetes Association report^21^ demonstrates this on a national scale, showing that Hispanic and NHB Medicaid beneficiaries are significantly less likely to use CGM compared to those covered by commercial insurance. Furtermore, in a qualitative study, using clinical vignettes to elicit CGM prescribing behavior with differences only in patient public or private insurance, Odugbesan et al.^17^ found that the majority of endocrinologists were less likely to recommend CGM in the public insurance vs. private insurance vignette.^17^ Although implicit bias cannot be ruled out, policy-level factors such as prior authorization complexity may play a more prominent role. Furthermore, limited access to digital resources and health information among publicly insured patients may make it more challenging for PCPs to initiate CGM.^22^ To support underserved patients in CGM use, practice transformations utilizing technology representatives for patient training and support, as well as partnerships with local pharmacies who can streamline prior authorization procedures, have been tested and shown to be highly feasible and effective.^23^

Language barriers appear to play a significant role, as Spanish-speaking patients were significantly less likely to receive CGM prescriptions compared to their English-speaking counterparts. Non-English speakers may experience challenges in receiving adequate information about CGM benefits and usage, potentially hindering technology acceptance. In addition, language support for printed materials, technology support lines, and other health literacy-associated barriers may not be adequately addressed with the current approaches to CGM marketing and practice implementation. Although Spanish-language CGM devices are now available, raising awareness among primary care teams can help ensure all patients benefit from these technologies. 

Older patients were less likely to receive CGM prescriptions, which may reflect provider or patient concerns about the ability of older adults to manage the technology. T1D Exchange Registry data has shown that, among 19,261 people with T1D, the likelihood of CGM use declined significantly after the age of 60,^16^ which is concerning due to the higher risk of hypoglycemia and hypoglycemia unawareness in this age group, that could be mitigated with CGM.^24,25^

Our study showed that HbA1c levels, insulin regimen intensity, and co-management with endocrinology increased the likelihood of PCP-prescribed CGM. In a study of approximately 3,000 US adults enrolled in Medicaid, Ni et al.^13^ similarly reported increased odds of CGM prescription among those with higher baseline HbA1c and on more intensive insulin regimens. This might reflect the actual or perceived need for intensive glucose monitoring in patients at higher risk of poor outcomes, and is a positive trend for CGM prescribing behavior that should be promoted and sustained. Ongoing education of primary care providers on clinical indications for CGM,^4^ recently expanded coverage criteria,^5^ and the demonstrated efficacy in HbA1c reduction for T2D patients using CGM may be helpful to continue to increase CGM adoption.^3,26,27^ Partnership with specialty care may lower barriers to CGM use in primary care by bolstering clinicians’ confidence and familiarity with device initiation.

Lastly, we were surprised to find that patients seen by more experienced providers were more likely to receive CGM prescriptions, given less proximity of their training to the release of newer diabetes technology. Similar to our results, Oser et al.^28^ showed that physicians-in-training were 70% less likely to prescribe CGM than non-trainee physicians, highlighting the gap in CGM education during residency. A nationwide survey^29^ of 134 family and internal medicine residency programs across the USA found that training on digital technologies in diabetes management was inadequate, with about 51% of respondents reporting minimal to no coverage of this topic in their curricula. This represents a significant opportunity to bridge gaps in trainee education on this standard of care diabetes management tool. CGM education for trainee physicians will be critical, as studies show that prior exposure substantially increased a clinician’s propensity to prescribe and recommend these technologies to patients.^28^

Our study has several limitations. First, this study was not able to explore certain patient-level factors such as personal preferences, refusal rates, or other reasons for not adopting CGM technology, which could have influenced CGM prescription rates. Although we adjusted for diabetes severity (complications, HbA1c trajectories, insulin regimen) and used insurance status as a proxy for income, the absence of measures like education, income, and diabetes duration means some residual confounding may persist. Further, prospective and qualitative study designs are needed to explore underlying causes. Nevertheless, this study’s strengths include the analysis of a large safety-net primary care network of over 1,200 primary care providers and 11,000 diverse patients; a robust longitudinal design using time-varying predictors to capture real-world changes in factors influencing CGM uptake over time; and the inclusion of multi-level factors to predict CGM prescription behavior. It is also important to note that there were no system-level efforts to enhance CGM prescribing in primary care during the study period, enabling us to accurately assess real-world prescribing behaviors without external interventions.

In conclusion, our findings show very low CGM prescription rates among eligible insulin-treated patients treated in primary care, despite generous insurance coverage for devices. Inequity in CGM disproportionately affected older adults, non-English speakers, and those with public insurance. By recognizing and addressing these factors that decrease the likelihood of CGM initiation and promoting increased awareness of current CGM prescribing guidelines, appropriate CGM prescribing may be improved. We have highlighted gaps in workflow efficiency, language and age-appropriate training support, and trainee education as key next steps to address in order to increase CGM prescriptions in primary care practice. Increasing CGM prescriptions is a critical first step to advancing diabetes management for underserved populations with diabetes. Future studies should explicitly test implementation strategies that address real-world barriers to CGM prescription and use, and evaluate clinical outcomes following primary care-initiated CGM prescription, to better understand the impact of CGM prescribing in these settings.

## Supplementary Information

Below is the link to the electronic supplementary material.

Supplementary Material 1 (DOCX 132 KB)
