# Supplementary material for: Inequity in Continuous Glucose Monitor (CGM) Prescribing Behaviors in Primary Care
Source: J Gen Intern Med. 2026 Jan 13;41(11):3041–8. doi: 10.1007/s11606-025-09923-7 (PMC13493726; doi:10.1007/s11606-025-09923-7)
Supplement: Supplementary file 1 [file 11606_2025_9923_MOESM1_ESM.docx]

**eMethods.** Multiple imputation for missing data

**Supplemental Table 1.** Proportion of missing HbA1c data over time

**Supplemental Table 2.** Baseline-to-follow up changes

**Supplemental Figure 1.** CGM prescribing rates

**Supplemental Table 3.** Cox regression sensitivity analysis

**eMethods. Multiple imputation for missing data**

HbA1c missing rate during study follow-up ranged from 1.3-21.8% (Supplemental Table 1). We used multiple imputation to estimate values of missing data. We used predictor variables that are known to affect HbA1c levels, and therefore we assumed that data were missing at random. The imputation model included available longitudinal HbA1c levels (all prior and later values for each individual), age, sex, BMI, microvascular, macrovascular complications, insulin treatment regimen and CGM prescription status. Predictive mean matching method was used to impute missing HbA1C levels and a total of 30 imputations were performed, based on a maximum of 20 iterations. The cause-specific Cox regression was then applied to the imputed datasets. Imputation was performed using the “mice” package (multivariate imputation via chained equations) and R version 4.3.2 (R Foundation for Statistical Computing). Sensitivity analyses using complete-case approach and a single last available HbA1c value were also performed (Supplemental Table 3).

| **Supplemental Table 1.** Proportion of missing HbA1c data over time | | | |
| --- | --- | --- | --- |
| **Months since study start** | **Number at risk** | **Cumulative number of censored patients** | **Percentage of time intervals with missing HbA1c levels** |
| 0 | 11,037 | 0 | 21.8 |
| 4 | 10,209 | 465 | 13.1 |
| 8 | 9,552 | 908 | 9.3 |
| 12 | 8,935 | 1,285 | 6.6 |
| 16 | 8,349 | 1,667 | 4.9 |
| 20 | 7,794 | 2,024 | 3.8 |
| 24 | 7,240 | 2,383 | 2.6 |
| 28 | 6,662 | 2,774 | 2.1 |
| 32 | 6,165 | 3,142 | 1.6 |
| 36 | 5,677 | 3,501 | 1.3 |

| **Supplemental Table 2.** Baseline-to-follow up changes | | | | | | | | |
| --- | --- | --- | --- | --- | --- | --- | --- | --- |
| Variable^a^ | **All** | | **No CGM** | | **CGM by PCP** | | **CGM by non-PCP** | |
|  | *Absolute*  *change^b^* | *Change,*  *%-pt^c^* | *Absolute*  *change^b^* | *Change,*  *%-pt^c^* | *Absolute*  *change^b^* | *Change,*  *%-pt^c^* | *Absolute*  *change^b^* | *Change,*  *%-pt^c^* |
| **Diabetes complications** | | | | | | | | |
| Any microvascular | +1023 | +9.3 | +821 | +10.0 | +130 | +6.9 | +72 | +7.6 |
| Any macrovascular | +1094 | +9.9 | +884 | +10.8 | +142 | +7.6 | +68 | +7.2 |
| Hypoglycemia event | +743 | +6.7 | +593 | +7.2 | +79 | +4.2 | +71 | +7.5 |
| **Hemoglobin A1c** **(mean)** | -0.8 | — | -1.0 | — | -0.1 | — | 0.0 | — |
| **Missing HbA1c** | -2189 | -19.8 | -1719 | -20.9 | -343 | -18.3 | -127 | -13.4 |
| **Insulin regimen** |  |  |  |  |  |  |  |  |
| Long‑acting insulin | +434 | +3.9 | +325 | +4.0 | +66 | +3.5 | +43 | +4.5 |
| Rapid‑acting insulin | +796 | +7.2 | +572 | +7.0 | +138 | +7.4 | +86 | +9.1 |
| Short‑acting insulin | +57 | +0.5 | +39 | +0.5 | +11 | +0.6 | +7 | +0.7 |
| Insulin NPH | +92 | +0.8 | +70 | +0.9 | +13 | +0.7 | +9 | +0.9 |
| Premixed insulin | +111 | +1.0 | +87 | +1.1 | +12 | +0.6 | +12 | +1.3 |
| **Endocrinology visit** | +1866 | +17.1 | +967 | +11.8 | +403 | +21.5 | +516 | +54.3 |
| Supplementary Table 2 summarizes characteristics that changed over time within each study group from baseline to end of follow-up. These variables were used as time-varying covariates in the Cox analysis (see Table 3).  ^a^Abbreviations: CGM - continuous glucose monitor; PCP - primary care provider; NPH - neutral protamine Hagedorn.  ^b^Absolute change represents the change in number of patients for categorical variables and the change in group mean for continuous variable.  ^c^%-pt change represents the absolute difference in prevalence (follow-up % − baseline %). | | | | | | | | |

**Supplemental Figure 1.** First-time CGM prescriptions by primary care providers (2020-2023)

Monthly CGM prescription rates were calculated by dividing the number of insulin-treated patients with type 2 diabetes who received a new CGM prescription from their primary care provider during a given month (and had no prior CGM use) by the total number of patients who had a primary care office visit that month and no history of CGM use. The yellow line represents the median prescription rate during study follow-up.\

| **Supplement Table 3.** Sensitivity analysis for missing HbA1c | | | | |
| --- | --- | --- | --- | --- |
| **Characteristic** | **Complete case analysis** | | **Last available HbA1c** | |
|  | **HR** | **95% CI** | **HR** | **95% CI** |
| Age |  |  |  |  |
| < 65 years |  |  |  |  |
| ≥ 65 years | 0.82 | 0.72, 0.92 | 0.83 | 0.73, 0.94 |
| Sex |  |  |  |  |
| Female | — | — | — | — |
| Male | 0.97 | 0.88, 1.06 | 0.96 | 0.88, 1.06 |
| Race-ethnicity |  |  |  |  |
| White | — | — | — | — |
| Hispanic | 0.98 | 0.76, 1.28 | 1.02 | 0.79, 1.32 |
| Black | 0.98 | 0.76, 1.26 | 1.00 | 0.78, 1.29 |
| Asian/Pacific | 0.80 | 0.55, 1.16 | 0.89 | 0.61, 1.29 |
| Other | 0.93 | 0.70, 1.24 | 0.99 | 0.74, 1.32 |
| Preferred language |  |  |  |  |
| English | — | — | — | — |
| Spanish | 0.72 | 0.63, 0.83 | 0.72 | 0.63, 0.84 |
| Other | 0.90 | 0.66, 1.22 | 0.87 | 0.64, 1.18 |
| Insurance |  |  |  |  |
| Commercial | — | — | — | — |
| Medicare | 0.88 | 0.76, 1.01 | 0.89 | 0.78, 1.03 |
| Dual | 0.84 | 0.70, 1.00 | 0.85 | 0.71, 1.02 |
| Medicaid | 0.67 | 0.58, 0.76 | 0.66 | 0.58, 0.75 |
| Any microvascular complication | 1.00 | 0.90, 1.12 | 1.06 | 0.95, 1.19 |
| Any macrovascular complication | 1.01 | 0.91, 1.12 | 1.04 | 0.94, 1.16 |
| Hypoglycemia event | 0.99 | 0.87, 1.13 | 1.00 | 0.88, 1.14 |
| Insulin regimen |  |  |  |  |
| Basal insulin only | — | — | — | — |
| MDI insulin | 1.42 | 1.28, 1.56 | 1.40 | 1.27, 1.54 |
| Hemoglobin A1c (per each %-pt increase) | 1.23 | 1.21, 1.26 | 1.30 | 1.28, 1.33 |
| Hospitalization during follow-up | 1.11 | 0.89, 1.39 | 1.13 | 0.90, 1.41 |
| Primary care office visit count (per each visit) | 1.07 | 1.06, 1.08 | 1.07 | 1.06, 1.08 |
| Endocrinology co-management | 1.10 | 0.99, 1.22 | 1.12 | 1.01, 1.24 |
| PCP years in practice (per each year) | 1.03 | 1.02, 1.04 | 1.03 | 1.02, 1.04 |
| PCP clinic |  |  |  |  |
| Family medicine | — | — | — | — |
| Internal medicine | 0.83 | 0.74, 0.93 | 0.81 | 0.73, 0.91 |
| *Abbreviations: CGM - continuous glucose monitor; MDI - multiple daily injection insulin; PCP - primary care provider.  Two sensitivity analyses addressed missing HbA1c values: a complete case analysis (n=1,800 events) and an approach using a single, last available HbA1c measure during follow-up (n=1,800 events). Adjusted HR and 95% CI were calculated using cause-specific Cox regression. Patients were censored at the time of first-time CGM prescription by non-primary care provider, death or at the end of follow-up. Time-varying covariates included microvascular, macrovascular complications, hypoglycemia events, insulin regimen changes, hemoglobin A1c levels, hospitalization events and primary care visit counts. Microvascular complications were defined as either neuropathy, retinopathy or nephropathy. Macrovascular complications were defined as cardiovascular, cerebrovascular or peripheral vascular disease. | | | | |
